# Nurses’ perceptions of the ISBAR handover protocol and its relationship to the quality of handover: A case study of bilingual nurses

**DOI:** 10.3389/fpsyg.2023.1021110

**Published:** 2023-02-23

**Authors:** Jack Pun

**Affiliations:** Department of English, The City University of Hong Kong, Kowloon, Hong Kong SAR, China

**Keywords:** nursing, handovers, communication, bilingual, perceptions

## Abstract

**Introduction:**

Poor communication at handover may cause harm to the patient. Despite numerous studies promoting ISBAR as a communication tool for structured handover, nurses have varied levels of understanding of the ISBAR tool; this may lead to different perceptions. This paper aims to explore the structural relationships between factors relating to handover communication among nurses.

**Method:**

A path analysis was conducted to analyse how 206 bilingual nurses’ knowledge of the ISBAR affects the perceived quality of handover, using a validated Nursing Handover Perception Questionnaire.

**Results:**

Nurses’ knowledge of the ISBAR was not a statistically significant factor affecting the perceived quality of handover. Rather, nurses’ understanding of patients’ care plans and receiving updated information about patients determine the perceived quality of handover.

**Discussion:**

Nurses’ compliance with the ISBAR tool should be considered in order to further identify and develop effective communication skills. Nurses’ understanding of patients’ care plans and receiving updated patient information significantly corresponded to the perceived quality of handover.

## Introduction

Clinical handover is defined as the “transfer of professional responsibility and accountability for some or all aspects of care for a patient, or a group of patients, to another person or professional group on a temporary or permanent basis” (Australian Medical Association, 2007). In nursing, the definition of a clinical handover is narrowed down to the process of transferring information about a patient’s condition and the responsibility of patient care to the nursing staff of another shift. It is recognised as a key component of clinical practice and is the most frequent and vital communication process occurring between nurses in the management of patient care (Eggins and Slade, 2015). During handover, nurses communicate with the nursing staff of the same or different wards, and sometimes with clinicians or other allied health professionals. Due to such complexity, the quality of communication at handover is crucial to a successful transfer of patient-care responsibility within and between specialists, wards, or care teams to ensure the continuity of effective care.

According to a study in one UK general hospital which detailed the most common types of handover incidents, unstructured handover is most commonly caused by the incompletion of the transfer process, including outdated or unclear patient forms, unsigned or missing drug charts, and an absence of a clear diagnosis and care plan (Pezzolesi et al., 2010). Additionally, in terms of communication, it may be caused by inadequate explanations about patient history and procedures to be done to the patient, faulty memory of the medical staff, absence of patients’ involvement, and more (Eggins and Slade, 2015). These issues may result in patient death; at the very least, they often lead to complaints.

Thus, establishing effective communication in healthcare practice has become a policy imperative worldwide. In bilingual hospital, where clinical staff normally communicate both first language and English for clinical information exchanges, this could lead to a complicated process of ‘code-switching’ or ‘translanguaging’ during clinical handover, which may be time-intensive, difficult to monitor and susceptible to errors (Pezzolesi et al., 2010). Nursing staff members working in a bilingual or multilingual society, such as those in Hong Kong, are required to fluently communicate in both English and in their first language for medical discussions and for their routine activities. Bilingualism in a medical environment is particularly complicated. Limited studies have investigated nursing handover practice in a bilingual context, and even fewer studies have explored nurses’ perceptions of handover protocols (e.g., ISBAR) and the possible factors affecting the perceived quality of handover in a bilingual context. Thus, this study explored bilingual nurses’ perceptions of the verbal communication tool, Introduction, Situation, Background, Assessment, and Recommendation (ISBAR) and evaluated its impact on the perceived quality of handover.

National and international health organisations have drawn attention to the importance of good-quality clinical handover for ensuring the well-being of patients, as patient safety is compromised in environments where information and responsibility are not adequately shared and conveyed during handover. The World Health Organization published an article on patient safety solutions, noting that problems in communication during clinical handover practices are universal (World Health Organization, 2007). Due to the prevalence of handover errors worldwide and their potential harm to patients, it is of paramount importance to constantly improve the quality of handovers.

Nurses play a significant role in patients’ daily care, and nurses’ communication and exchange of information about patients’ conditions is crucial to sustaining good-quality patient care. Comprehensive and effective transfer of patient care responsibility during clinical handover is therefore critical. Nurses between shifts and across different disciplinary teams or clinical units should take an active role in enhancing the effectiveness of communication at handover, as studies show that structured and consistent handover methods, such as a checklist, could vastly improve the quality of handover and minimise handoff-related care failures (Boat and Spaeth, 2013; Bigham et al., 2014; Ferorelli et al., 2017). Over recent decades, an increasing number of studies have consistently pointed out that poor clinical handover contributes to many incidents that result in avoidable patient harm and medical failures (Nakajima et al., 2005; Raeisi et al., 2019). Poor clinical handovers also create discontinuities in care, which lead to patient harm. For instance, Foster and Manser noted that clinical handover is a vulnerable point in a patient’s care and that ineffective communication during handover did lead to mistakes or loss of information (Foster and Manser, 2012). Other researchers have stated that if the handover is unstructured or inconsistent, information exchanged between staff about patients may be incomplete, leading to workflow inefficiencies and obstruction of the ability of staff to monitor patients and provide suitable care (Arora et al., 2005; Methangkool et al., 2019). In emergency care, unstructured handover also hinders prioritisation and patient disposition practitioners cannot solely depend on documentation, as verbal communication is just as important; poor handover would also hinder prioritisation and patient disposition, causing the inability to maximize patient flow (Sujan et al., 2014). Furthermore, the nature of clinical handovers includes many different stakeholders with varying responsibilities and is highly dependent on communication; the handover practice then becomes more difficult to standardise (Jeffcott et al., 2009; Göbel et al., 2012).

Thus, without a standardised and validated communication protocol, nurses may find it challenging to communicate effectively regarding clinical procedures and patients’ conditions, leading to a lack of shared understanding of their patients’ conditions and the creation of an unsafe clinical environment (Leonard et al., 2004). In recent years, an increasing number of nursing studies have focused on developing interventions for promoting effective handover using standardised communication tools as part of protocols, such as the Identify, Situation, Background, Assessment and Recommendation (ISBAR) tool, for structuring handover practices (Finnigan et al., 2010; Flemming and Hübner, 2013; Robertson et al., 2014; Kitney et al., 2016). Instead of a sole regime being imposed on the medical staff, study has shown than they also believe ISBAR is helpful in promoting presentations that are more prioritised and eliciting the crucial elements of handovers. Many of these studies have illustrated that the use of the ISBAR tool assisted nurses in establishing structured communication at handover and presenting information in a logical manner. The use of ISBAR would in turn reduce the loss of vital information, miscommunication, and misunderstandings, as well as increase the possibility of timely and efficient handover being conducted among staff members (Mannix et al., 2017).

Sandlin, a nurse manager in outpatient surgical services, suggested that the ISBAR tool could greatly improve patient safety (Sandlin, 2007). Specifically, it enables the caregiver who is taking the report an opportunity to read back, repeat back, and ask questions. Complete and concise handover communication following a validated format will improve the quality of the handover. However, other studies have shown contrasting findings, as nurses using the same protocol may have different perceptions of their handover practices, resulting in misalignment between what the nurses said and the actual structure of the communication of the information (Pun et al., 2019, 2020). In one study, even experienced nurses who used a standardised checklist did not exhibit a high level of consistency in handovers (Eggins et al., 2016). In addition, a study by Chiew et al. demonstrates that nurses’ perceptions of and compliance with the ISBAR tool are essential to achieving effective communication, yet studies on the relationship between perception and compliance are lacking (Chiew et al., 2019). It is thus necessary to evaluate the aforementioned relationship.

Research exploring handover practices and the use of the ISBAR protocol among nurses in a bilingual context is very limited. The available studies are from Hong Kong (Pun et al., 2019, 2020), wherein a training programme was delivered using the ISBAR protocol to promote the effectiveness of communication among nurses at handovers. Their findings suggest that better training of nurses in handover practice enhances nurses’ perceptions and understanding of the ISBAR protocol and likely improves patient safety and the continuity of care. A study done in Qatar on a similar tool, the SBAR, revealed that over 87% of their nurses believed that the SBAR was good and that over 95% of these nurses use the SBAR tool during handover most of the time or always (Nagammal et al., 2016). This suggests that nurses’ positive perception of the tool has a corresponding impact on how frequently this tool is used. According to a study on the perceived effectiveness of the ISBAR tool (Thompson et al., 2011), after the introduction of ISBAR, 71% of the medical staff involved believed that the tool improved handover and 80% felt more confident about their handover skills. More efficient communication of clinical information is evident when the ISBAR is used. Staff members also believed that handover was more consistent, better structured, and of a higher quality with the use of ISBAR. This shows that staff members’ positive perceptions of handover tools increases may increase their usage of the ISBAR in their clinical practice, hence possibly affecting the quality of handover.

Aside from a consistent structure, a good quality nursing handover should take place in a quiet and low-stress environment, and nurses should have a clear understanding of the patient’s status, adequate opportunities to conduct a dialogue that allows nurses to ask questions without interrupting the structure of the handover, and the ability to receive updated information about patients (Loefgren Vretare and Anderzén-Carlsson, 2020). Therefore, this study aimed to explore the structural relationships between factors relating to nurses’ handover communication.

## Methods

### Aims

This study aims to examine the structural relationships between the factors that may relate to nurses’ handover communication. These factors are nurses’ knowledge/perception of the ISBAR, the perceived quality of handover, receiving up-to-date information, and understanding of the patient care plan.

### Participants

Participants in this research were nursing staff from a local hospital in Hong Kong. All participants work in a bilingual context involving both English and Cantonese.

### Data collection

An invitation letter of communication training was sent to each nursing staff in the hospital. Those who agreed to attend the training were asked to submit a written consent form with their own signature. Subsequently, 206 participants joined our training program in 2017–18. A communication training program was conducted for nursing staff as a platform for recruiting participants using convenient sampling technique. Researchers conducted a paper-and-pencil survey aimed at evaluating the perceived effectiveness of communication training on handover among nursing staff. A validated Nurses’ Handover Perceptions Questionnaire (NHPQ) was adopted for the survey.

### Ethical considerations

The ethical review board of the participating hospital approved this study. All participants received a verbal explanation of the aims and design of the research project, as well as their right to withdraw at any time and an assurance of confidentiality. Written informed consent was obtained from all participants during each phase of the project. All methods were carried out in accordance with relevant guidelines and regulations.

### Validity, reliability, and rigour

The survey was originally adapted from the scales used by Klim, Kelly, Kerr, Wood and McCann (Klim et al., 2013) and Street, Eustace, Livingston, Craike, Kent, and Patterson (Street et al., 2011) to identify nurses’ perceptions of their current practices and of the components essential for effective shift-to-shift nursing handovers. It has been validated in a Hong Kong-based study that evaluated nurses’ perceptions of and communication practices during handovers (Pun et al., 2019, 2020), where it was shown to have a high degree of reliability (Cronbach’s alpha = 0.99) and an intra-class correlation coefficient of 0.92.

The final version of the questionnaire includes 23 items centred on nurses’ perceptions of the presentation, organisation, comprehension, and dissemination of patient information and their knowledge of the ISBAR protocol. To reduce possible response bias and simplify the analysis, all of the survey items were rated on a 4-point Likert scale from 1 to 4, with 1 indicating ‘strongly disagree’, 2 – ‘disagree’, 3 – ‘agree’, and 4 – ‘strongly agree’.

Five out of the 23 items were rated on four variable measures in this study, namely the knowledge of the ISBAR protocol, perceived quality of handover, up-to-date information about the patient, and understanding of the patient care plan. Specifically, the perceived quality of handover was measured by an item on whether the handover information was presented in a systematic and organised manner; up-to-date information referred to the item asking about the amount of updated information about patients that was received by nurses after the training; understanding of the patient care-plan is assessed by the item on participants’ knowledge of diagnosis, treatment, and discharge about the patients after training. To measure nurses’ knowledge/perception of the ISBAR protocol, two items, namely (a) ‘I believe that using ISBAR will help me improve my communication skills with co-workers’ and (b) ‘I believe that using ISBAR will increase patient safety and care quality’ were computed into a mean score, and the internal consistency (Cronbach’s alpha = 0.92) was deemed acceptable.

### Data analysis

**Figure 1:**
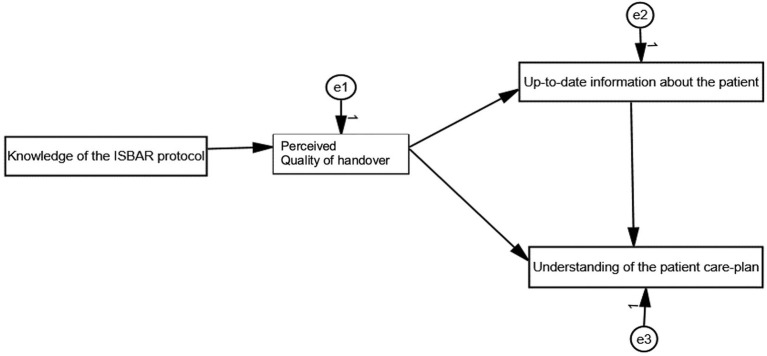
Hypothesised model.

To ensure the validity of the analysis, collected questionnaires with more than 10% missing data were excluded during the analysis (Dong and Peng, 2013). Descriptive statistics of demographic information and correlations between the variables were evaluated using SPSS 21.0. In addition, a path analysis, which aimed to explain structural connections between the variables in a hypothesized model (Figure 1) was performed using the AMOS 21.0 program. A chi-square statistic (*χ*^2^), Steiger’s root-mean-square error of approximation (RMSEA), the Tucker–Lewis index (TLI), and a comparative fit index (CFI) were used to describe the model fit. The Tucker–Lewis index, CFI, and RMSEA were used in addition to *χ*^2^, as the latter is very sensitive to sample size. Generally, RMSEA values of ≤0.05 and TLI and CFI values of >0.9 are considered to indicate a reasonably good model fit (Blunch, 2013). The hypothesised model is presented in Figure 1.

## Results

Table 1 presents the descriptive statistics of the analysis. Over 90% of the participants were female (*n* = 186, 90.3%), and most had a working experience of more than 6 years (*n* = 135, 65.6%). The majority of the participants were aged from 30 to 39 years (*n* = 79, 38.3%). Approximately half of the 206 respondents (*n* = 100, 48.5%) had a Bachelor’s degree and 35.5% (*n* = 73) had a Master’s degree or above.

**Table 1:** Descriptive statistics.

		*n*	Percentage (%)
Gender	Female	186	90.3
	Male	14	6.8
	Missing data	6	2.9
Age	20–29	69	33.5
	30–39	79	38.3
	40–49	41	19.9
	50 or above	14	6.8
	Missing data	3	1.5
Education	Diploma	33	16
	Bachelor	100	48.5
	Master or above	73	35.5
Working experience in current hospital (year)	0–1	12	5.8
	2–5	59	28.6
	6–10	71	34.5
	> = 10	64	31.1

Table 2 shows the bivariate correlations between the factors shown in the hypothesised model. Except for the knowledge of the ISBAR protocol, all variables were significantly correlated with each other. Specifically, understanding of the patient care plan was significantly correlated with the perceived quality of handover and up-to-date information that nurses received during the handover.

**Table 2:** Bivariate correlations between factors.

	1	2	3	4	5
Perceived quality of handover	1				
Up-to date information about the patient	0.17*	1			
Understanding of the patient care-plan	0.24**	0.39**	0.38**	1	
Knowledge of the ISBAR protocol	0.05	0.10	−0.03	0.01	1

*p < 0.05, **p < 0.01.

**Figure 2:**
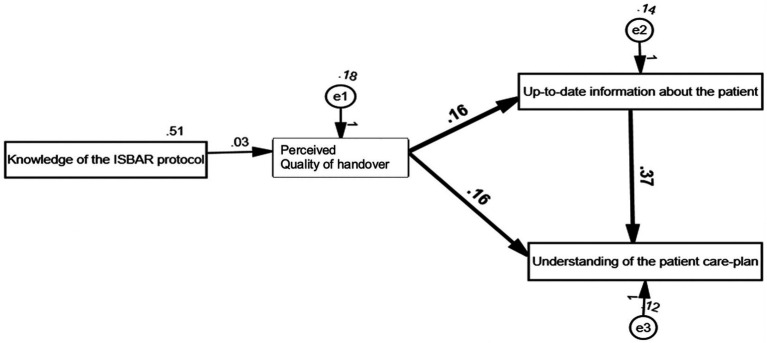
Hypothesised model with path coefficients.

The indices of the hypothesised model indicated a very good model fit [*χ*^2^(2) = 0.93, *p* > 0.05, RMSEA = 0.00, TLI = 1.01, CFI = 1.00; Figure 2]. As shown in Table 3, three of the hypothesised paths were statistically significant. The perceived quality of handover was found to be directly connected with the understanding of the patient care plan and the level of up-to-date information about the patient that they received. In addition, up-to-date information was positively and significantly associated with the understanding of the patient care plan. However, no significant association was found between participants’ knowledge of the ISBAR protocol and the perceived quality of handover.

**Table 3:** Path coefficients.

Model path	Unstandardised coefficient (S.E.)	Standardised coefficient
Knowledge of the ISBAR protocol → quality of handover	0.03 (0.04)	0.05
Perceived quality of handover → up-to date information about the patient	0.16 (0.06)*	0.17
Perceived quality of handover → understanding of the patient care-plan	0.16 (0.06)**	0.17
Up-to date information about the patient → understanding of the patient care-plan	0.37 (0.07)***	0.36

*p < 0.05, **p < 0.01, ***p < 0.001.

## Discussion

This study focuses on identifying structural relationships between the factors that may associate to nurses’ handover communication skills, which are nurses’ knowledge/perception of the ISBAR, the perceived quality of handover, receiving up-to-date information, and understanding of the patient care plan.

As the findings show, the perceived quality of handover was found to be directly associated with understanding of the patient care plan and the level of up-to-date information they received about the patient. In addition, up-to-date information about the patient during handover was significantly associated with enhancing understanding of the patient care plan. No strong connection was found between participants’ perception of the ISBAR protocol and the perceived quality of handover.

**Figure 3:**
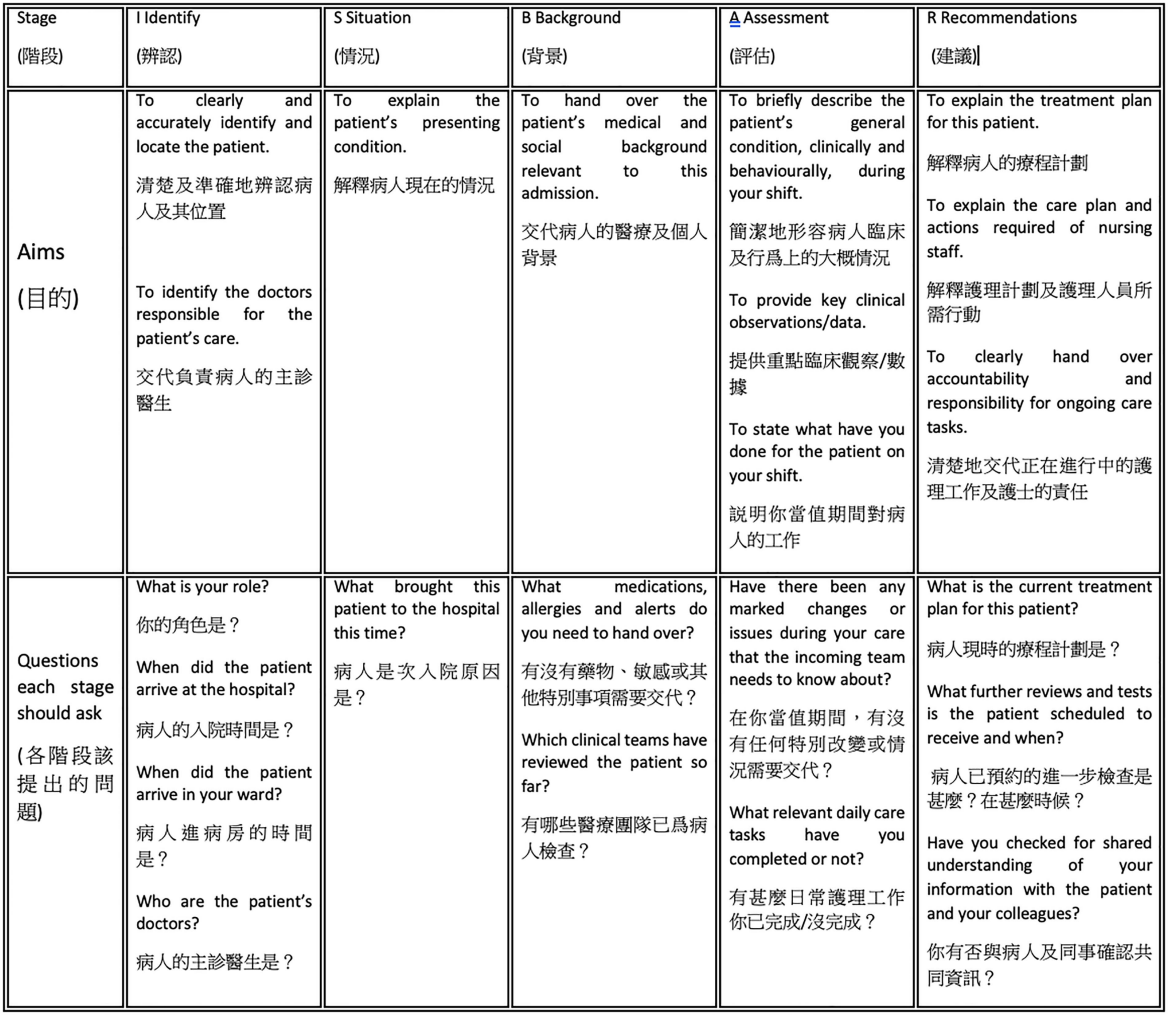
The ISBAR protocol for bilingual nursing handover.

The adapted ISBAR as a verbal communication tool (see Figure 3) stands for five main stages of a structured nursing handover practice that is intended to help to improve communication among nurses and avoid patient harm, namely Identify, Situation, Background, Assessment and Recommendation. It is a validated tool that aims to assist nurses, in particular those working in a multicultural environment, in establishing and preparing structured communication by guiding the actions to be taken during and after handover. It also helps nurses present information in a logical manner. In Hong Kong, the ISBAR protocol has been used in clinical wards for many years, yet few studies have evaluated how a bilingual nurse’s perceptions of the ISBAR can affect the perceived quality of handover within a bilingual context. In this study, nurses’ knowledge of the ISBAR was not a statistically significant factor to predict their perceived quality of handover. Instead, their understanding of patients’ care plans and level of receiving updated patient’s information determine the perceived quality of handover. Nevertheless, since this study did not measure the actual degree of using ISBAR by nursing staff, the effectiveness of ISBAR training in terms of improving nurses’ perceived quality of handover skills remains unknown. To further evaluate the adapted ISBAR tool in the future, a possible approach would be using simulation as simulation has proven to be essential in testing and long-term education for nurses^b^. Research also has shown that the adaption of simulation could contribute to nurses’ preparedness for scenarios in real life^c^.

**Figure 4:**
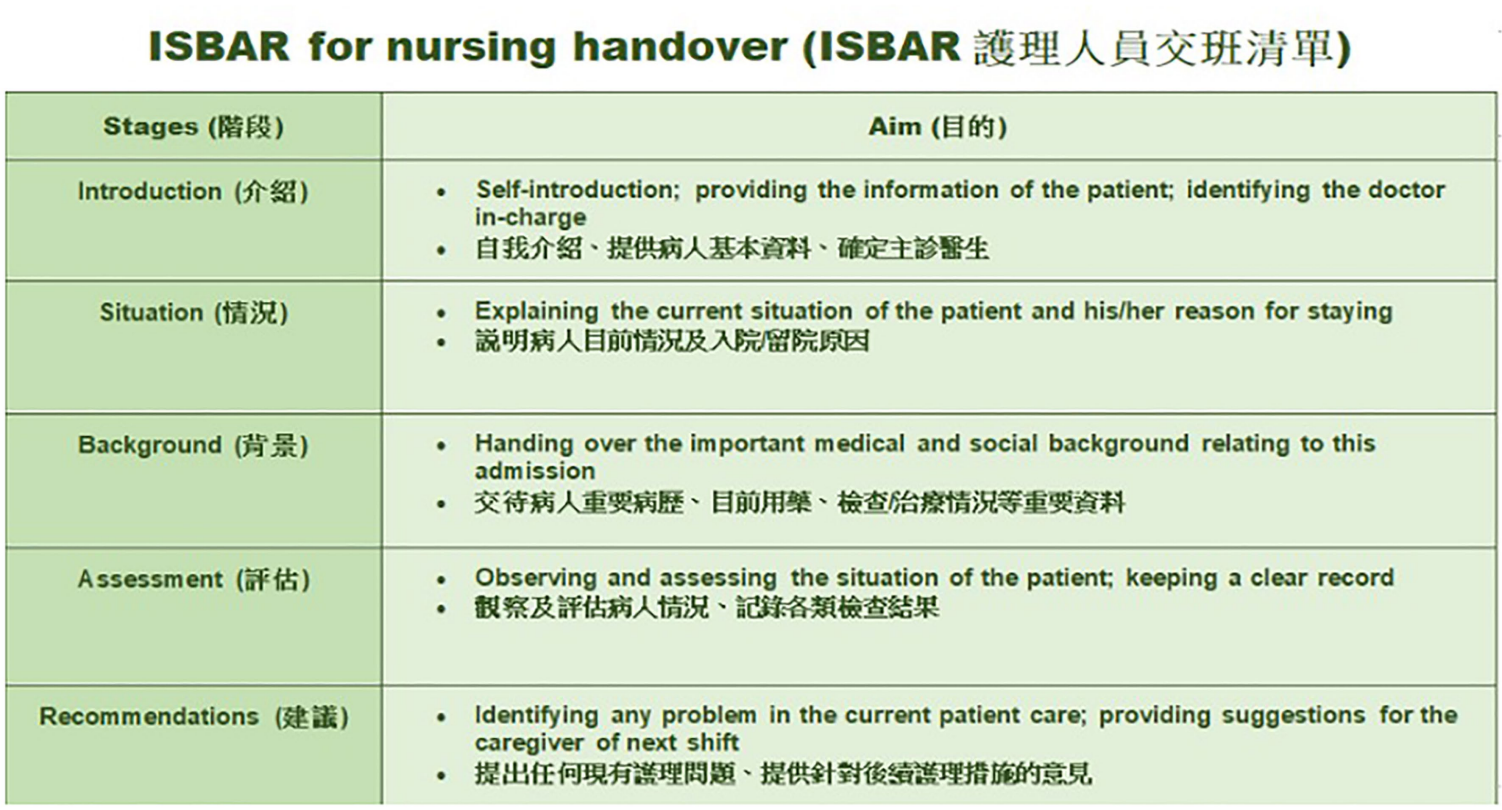
Bilingual version of the ISBAR protocol for nursing handover.

In Hong Kong, the use of Cantonese for reporting patients’ latest condition, together with the use of English for communicating medication or treatment information, code-mixing between Cantonese and English at handovers, and the use of English for medical records and in the training of healthcare practitioners make the bilingual handover communication highly complex (Bourhis et al., 1989). This complexity in a bilingual context is especially evident among nurses with less experience. This may create many opportunities for loss of information during handover communications, which is why a bilingual version of the ISBAR protocol (see Figure 4) should be made available to enable bilingual nurses to conduct an effective and accurate exchange of information about patients’ conditions in both Cantonese and English to ensure good-quality patient care. It is believed that training in the use of the ISBAR tool would reduce the frequency and severity of patient harm resulting from poor quality handover and improve teamwork within and across disciplines (Bakon et al., 2017).

Notably, the findings of this study show that the perceived quality of handover affects not only a nurse’s understanding of the patient care plan but also the level of up-to-date information being received. In the bilingual context in Hong Kong, it is important that these two factors are consistent in both written and spoken versions to ensure information accuracy and to maintain a high level of understanding between incoming and outgoing nurses. Hence, improving the quality of handover is crucial to medical service for patient treatment. Since the education level of nurses varies, switching between two different languages may lead to different handover outcomes (Rossiter et al., 1998), which may probably lead to a misunderstanding during an information delivery. In this regard, hospital administrators and policymakers should pay attention to emphasize the importance of handover and provide continuous training for improving the nursing staff’s systematic communication skills.

This study has some limitations. First, participants were nurses from only single local hospital in Hong Kong, therefore the research findings should be generalised with caution. Second, though the results provide meaningful insights about clinical handovers, solely considering nurses’ perceptions of ISBAR, rather than their actual practices, which is not enough to explain its effectiveness. As aforementioned, there should be a gap between perceptions and actual practices. Thus, further research is warranted to explore more corresponding factors that may influence the effects of ISBAR training on nurses’ handover skills.

The ISBAR protocol has been used for better structured clinical handovers for many years. In a bilingual context, such as in Hong Kong hospitals, communication failure may often occur because of the difficult and complex switches between medical language (English) and everyday language (Cantonese). The findings show that bilingual nurses’ perception of the ISBAR tool and the perceived quality of handover are not directly related, but that the perceived quality of handover is more related to these bilingual nurses’ understanding of the patient care-plan and receiving of up-to-date information from outgoing nurses. For further improvements to be made in clinical practice, professional training programmes for bilingual nurses should aim to increase nurses’ ability to communicate updated patient care-plans and their ability to understand patient information in both English and Cantonese.

## Conclusion

This study focused on nurses working in a bilingual environment and investigated the structural relationships between factors that may connect to their handover communication. The results show that the nurses’ perceptions of the ISBAR protocol do not directly predict their handover performance. However, handover quality is significantly related to nurses’ knowledge of patients’ care plans and receipt of updated information about patients’ conditions, which are essential to high-quality healthcare service. Though nurses’ knowledge of the ISBAR may not be a driving force to directly change their perceived quality of handover, participants’ compliance with ISBAR should be considered as well for further research to identify its effectiveness in developing nurses’ handover communication skills. Especially in a multicultural context, nursing training programmes should be continuously provided to fulfil bilingual nurses’ ability to deliver and understand sufficient patient information.

## Data availability statement

The raw data supporting the conclusions of this article will be made available by the authors, without undue reservation.

## Ethics statement

The studies involving human participants were reviewed and approved by Hong Kong Sanatorium and Hospital ethics committee. The patients/participants provided their written informed consent to participate in this study.

## Author contributions

JP contributed to the conception, design, data collection, analysis and interpretation of data, writing, and revising the manuscript.

## Conflict of interest

The author declares that the research was conducted in the absence of any commercial or financial relationships that could be construed as a potential conflict of interest.

## Publisher’s note

All claims expressed in this article are solely those of the authors and do not necessarily represent those of their affiliated organizations, or those of the publisher, the editors and the reviewers. Any product that may be evaluated in this article, or claim that may be made by its manufacturer, is not guaranteed or endorsed by the publisher.

## Abbreviations

ISBAR: Introduction, Situation, Background, Assessment
ISBARQ: Introduction, Situation, Background, Assessment, Recommendation and Question and answer
NHPQ: Nurses Handover Perceptions Questionnaire
RMSEA: Root mean square error of approximation
CFI: Comparative fit index
TLI: Tucker–Lewis index
